# Mutation of von Hippel–Lindau Tumour Suppressor and Human Cardiopulmonary Physiology

**DOI:** 10.1371/journal.pmed.0030290

**Published:** 2006-06-20

**Authors:** Thomas G Smith, Jerome T Brooks, George M Balanos, Terence R Lappin, D. Mark Layton, Dawn L Leedham, Chun Liu, Patrick H Maxwell, Mary F McMullin, Christopher J McNamara, Melanie J Percy, Christopher W Pugh, Peter J Ratcliffe, Nick P Talbot, Marilyn Treacy, Peter A Robbins

**Affiliations:** 1Department of Physiology, Anatomy, and Genetics, University of Oxford, Oxford, United Kingdom; **2**School of Sport and Exercise Sciences, University of Birmingham, Birmingham, United Kingdom; **3**Centre for Cancer Research and Cell Biology, Queen's University, Belfast, United Kingdom; **4**Department of Haematology, Imperial College of Science, Technology, and Medicine, London, United Kingdom; **5**Diagnostics, Therapies, and Cancer Division, Chase Farm Hospital, Middlesex, United Kingdom; **6**Renal Section, Imperial College of Science, Technology, and Medicine, London, United Kingdom; **7**Department of Haematology, Queen's University, Belfast City Hospital, Belfast, United Kingdom; **8**Department of Haematology, Royal Free Hospital, Hampstead, United Kingdom; **9**Nuffield Department of Clinical Medicine, University of Oxford, Oxford, United Kingdom; National Heart and Lung InstituteUnited Kingdom

## Abstract

**Background:**

The von Hippel–Lindau tumour suppressor protein–hypoxia-inducible factor (VHL–HIF) pathway has attracted widespread medical interest as a transcriptional system controlling cellular responses to hypoxia, yet insights into its role in systemic human physiology remain limited. Chuvash polycythaemia has recently been defined as a new form of VHL-associated disease, distinct from the classical VHL-associated inherited cancer syndrome, in which germline homozygosity for a hypomorphic VHL allele causes a generalised abnormality in VHL–HIF signalling. Affected individuals thus provide a unique opportunity to explore the integrative physiology of this signalling pathway. This study investigated patients with Chuvash polycythaemia in order to analyse the role of the VHL–HIF pathway in systemic human cardiopulmonary physiology.

**Methods and Findings:**

Twelve participants, three with Chuvash polycythaemia and nine controls, were studied at baseline and during hypoxia. Participants breathed through a mouthpiece, and pulmonary ventilation was measured while pulmonary vascular tone was assessed echocardiographically. Individuals with Chuvash polycythaemia were found to have striking abnormalities in respiratory and pulmonary vascular regulation. Basal ventilation and pulmonary vascular tone were elevated, and ventilatory, pulmonary vasoconstrictive, and heart rate responses to acute hypoxia were greatly increased.

**Conclusions:**

The features observed in this small group of patients with Chuvash polycythaemia are highly characteristic of those associated with acclimatisation to the hypoxia of high altitude. More generally, the phenotype associated with Chuvash polycythaemia demonstrates that VHL plays a major role in the underlying calibration and homeostasis of the respiratory and cardiovascular systems, most likely through its central role in the regulation of HIF.

## Editors' Summary

**Background.:**

Human cells (like those of other multicellular animals) use oxygen to provide the energy needed for daily life. Having not enough oxygen is a problem, but having too much is also dangerous because it damages proteins, DNA, and other large molecules that keep cells functioning. Consequently, the physiological systems—including the heart, lungs, and circulation—work together to balance oxygen supply and demand throughout the body. When oxygen is limiting (a condition called hypoxia), as happens at high altitudes, the cellular oxygen supply is maintained by increasing the heart rate, increasing the speed and depth of breathing (hyperventilation), constricting the blood vessels in the lung (pulmonary vasoconstriction), and increasing the number of oxygen-carrying cells in the blood. All these physiological changes increase the amount of oxygen that can be absorbed from the air, but how they are regulated is poorly understood. By contrast, researchers know quite a bit about how individual cells respond to hypoxia. When oxygen is limited, a protein called hypoxia-inducible factor (or HIF) activates a number of target proteins that help the cell get enough oxygen (for example, proteins that stimulate the growth of new blood vessels). When there is plenty of oxygen, another protein, called von Hippel–Lindau tumor suppressor (abbreviated VHL), rapidly destroys HIF. Recently, researchers discovered that a genetic condition called Chuvash polycythaemia, characterised by the overproduction of red blood cells, is caused by a specific defect in VHL that reduces its ability to destroy HIF. As a result, the expression of certain HIF target proteins is increased even when oxygen levels are normal.

**Why Was This Study Done?:**

Chuvash polycythaemia is very rare, and so far little is known about how this genetic abnormality affects the physiology and long-term health of patients. By studying heart and lung function in patients with Chuvash polycythaemia, the researchers involved in this study hoped to discover more about the health consequences of the condition and to find out whether the VHL–HIF system controls systemic responses to hypoxia as well as cellular responses.

**What Did the Researchers Do and Find?:**

The researchers recruited and studied three patients with Chuvash polycythaemia, and, as controls for the comparison, several normal individuals and patients with an unrelated form of polycythaemia. They then measured how the lungs and hearts of these people reacted to mild hypoxia (similar to that experienced on commercial air flights) and moderate hypoxia (equiv alent to being on the top of an Alpine peak). They found that patients with Chuvash polycythaemia naturally breathe slightly quicker and deeper than normal individuals, and that their breathing rate increased dramatically and abnormally when oxygen was reduced. They also found that at normal oxygen levels the pulmonary blood vessels of these patients were more constricted than those of control individuals, and that they reacted more extremely to hypoxia. Similarly, the normal heart rate of the patients was slightly higher than that of the controls and increased much more in response to mild hypoxia.

**What Do These Findings Mean?:**

The physiological differences measured by the researchers between Chuvash polycythaemia patients and control individuals are similar to the adaptations seen in people traveling to high altitudes where oxygen is limited. Thus, the VHL–HIF proteins may regulate the response to different oxygen concentrations both in individual cells and at the systemic level, although more physiological studies are needed to confirm this. Because the pulmonary blood vessels of patients with Chuvash polycythaemia are always abnormally constricted, and even more so when oxygen is limited, these people should avoid living at high altitude and should minimise air travel, suggest the researchers. The increased blood pressure in their lungs (pulmonary hypertension) could conceivably cause heart failure under such circumstances. Finally, this study has implications for the development of drugs directed at the VHL–HIF system. Agents are currently being designed to promote the development of new blood vessels after strokes or heart attacks by preventing the destruction of HIF, but based on the findings here such agents might have undesirable physiological affects. Conversely, HIF inhibitors (which act as anti-cancer reagents by increasing hypoxia in the centre of tumors and so inhibiting their growth) might be useful in the treatment of pulmonary hypertension.

**Additional Information.:**

Please access these Web sites via the online version of this summary at http://dx.doi.org/10.1371/journal.pmed.0030290.

• Online Mendelian Inheritance in Man page on Chuvash polycythaemia

• Information from the VHL Family Alliance on von Hippel–Lindau disease, including information on Chuvash polycythaemia

• Wikipedia page on polycythaemia and von Hippel–Lindau disease (note: Wikipedia is a free online encyclopaedia that anyone can edit)

## Abstract 3

Physiological study of patients with Chuvash polycythemia (caused by mutation of VHL) reveals characteristics similar to those associated with acclimatization to the hypoxia of high altitude.

## Introduction

Hypoxia is universally experienced at high altitudes and is a detrimental feature of many medical conditions. The human response to acute hypoxia includes hyperventilation, pulmonary vasoconstriction, systemic peripheral vasodilation, and tachycardia. Our understanding of how these systemic responses are evoked and regulated remains limited. However, it is now known that intracellular responses to hypoxia are coordinated by the hypoxia-inducible factor (HIF) family of transcription factors, which directly or indirectly regulate the expression of several hundred genes in any given cell type [ 1]. The von Hippel–Lindau tumour suppressor protein (VHL) is an essential component in the degradation pathway through which HIF is primarily regulated [ 2, 3]. The HIF-α subunit is synthesised continuously but is rapidly destroyed in the presence of oxygen. Oxygen-dependent prolyl hydroxylases hydroxylate specific residues in HIF-α, increasing its affinity for VHL [ 4, 5]. The binding of VHL to hydroxylated HIF-α then targets HIF-α for destruction by the ubiquitin–proteasome pathway [ 2, 3]. Under hypoxic conditions, the hydroxylation of HIF-α is inhibited, proteasomal degradation is slowed, and thus there is rapid accumulation of HIF with subsequent up-regulation of hypoxia-responsive genes. Manipulation of the VHL–HIF pathway is currently under intense investigation as a treatment for cancer and ischaemic/hypoxic vascular disease [ 6]. However, the role of the VHL–HIF pathway in systems-level human physiology remains largely unknown.

In the classical VHL-associated inherited cancer syndrome, affected individuals are heterozygous for a germline VHL mutation that predisposes to specific types of tumour [ 7]. Known clinical manifestations are confined to tumours and discrete benign lesions that arise following somatic inactivation of the second allele. Disturbance of hypoxia signalling is limited to tumour cells, and studies of these effects have been confined to the phenotype of associated renal cell carcinoma and to lesions that develop in rodent models of VHL inactivation. Such analyses essentially examine the effects of complete dysregulation of the VHL–HIF system at the cellular level.

Chuvash polycythaemia (CP) is a rare autosomal recessive disorder caused by an entirely distinct disease mechanism. Affected individuals are homozygous for a specific *598C>T* mutation in VHL [ 8] that impairs but does not ablate the regulation of HIF—potentially allowing insights into its systemic functions in humans. The VHL mutation diminishes VHL's binding affinity for hydroxylated HIF-α, thereby partially inhibiting HIF-α degradation and pathologically up-regulating HIF target genes including *erythropoietin (EPO)* [ 8]. CP is characterised by congenital erythrocytosis, but has yet to be extensively phenotyped. Through experiments conducted with CP patients, this study aimed to investigate the potential role of the VHL pathway in cardiopulmonary physiology. It has demonstrated profound abnormalities in ventilatory and pulmonary vascular control in affected individuals.

## Methods

### Participants

Physical characteristics of the participants are given in Table 1. Patients with CP were identified from a previous study [ 9] and recruited through their respective consultant haematologists. Each patient was homozygous for the classic Chuvash mutation and had been treated with long-term venesection to maintain a normal haemoglobin and haematocrit. No patient had been venesected within several weeks of the experiment. They had no other medical disorders, had no history of complications, and were asymptomatic except for occasional headaches. The size of the CP patient group was limited by the rarity of the condition in the United Kingdom. The normal control group comprised six age- and sex-matched healthy volunteers who were recruited by advertisement. The CP patient group (age 22.3 ± 5 y, mean ± SD) and the normal control group (24.2 ± 5 y) did not differ significantly in age, height, weight, or body mass index. A further three patients were recruited as sex-matched polycythaemia control participants: two men diagnosed with acquired idiopathic erythrocytosis and one woman with polycythaemia vera. No mutations were detected on sequencing the VHL gene in these patients. Each was otherwise in good health and had been chronically venesected to a normal hematocrit. These patients were somewhat older than the CP patient group ( Table 1) but were otherwise similar. The female polycythaemia control participant was taking daily aspirin and citalopram, and one male polycythaemia control was taking daily atenolol, aspirin, and simvastatin for cardiovascular disease prophylaxis. Otherwise, participants were taking no medications, vitamin supplements, or caffeine. The study had been approved by the Oxfordshire Clinical Research Ethics Committee, and each participant provided written informed consent.

**Table 1:**
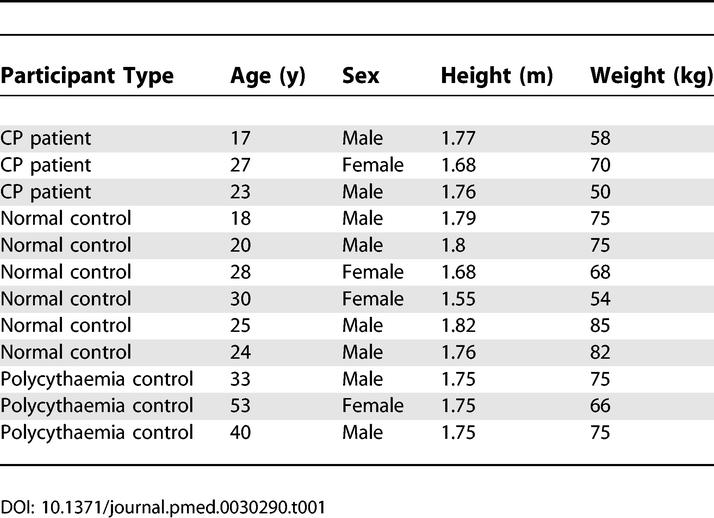
Participant Characteristics

### Studies of HIF-Regulated Gene Expression

These studies were undertaken to confirm differences in HIF-regulated gene expression at the mRNA level in CP patients compared with normal controls. Mononuclear cells were isolated by centrifugation of venous blood over a density gradient medium (Ficoll-Paque Plus, Amersham Biosciences, Chalfont, United Kingdom). The mononuclear cells were resuspended in 40 ml of RPMI 1640 cell culture medium supplemented with 10% fetal bovine serum, L-glutamine, and penicillin/streptomycin antibiotics (Sigma-Aldrich, Gillingham, United Kingdom). The cells were placed in tissue culture dishes with a hydrophilic base (Vivascience, Hannover, Germany) at 37 °C for 30 min to allow for adherence of the monocytes to the base. The supernatant containing the lymphocytes was then removed and aliquotted into eight tissue culture dishes in which the base was both hydrophobic and gas permeable (Vivascience). Lymphocytes were incubated for 20 h at 37 °C in 5% carbon dioxide and at one of eight different oxygen tensions (20%, 10%, 5%, 2%, 1%, 0.5%, 0.2%, or 0.1%). The cells were then harvested and RNA was isolated using a column-based centrifugation extraction protocol (RNAqueous-4PCR; Ambion-Europe, Huntingdon, United Kingdom). RNA samples were reverse transcribed using random hexamer primers, and the HIF-regulated genes *Aldolase C* and *vascular endothelial growth factor (VEGF)* were quantified by real-time quantitative PCR using Taqman primers and probes (Applied Biosystems, Warrington, United Kingdom). The gene *β _2_-microglobulin* was used as an internal experimental control and was quantified by the same method. A standard calibrator sample of cDNA was assayed for these three genes with each set of real-time quantitative PCR reactions. This sample was derived from the pooled RNA of two 20-h incubations of lymphocytes at 20% and 0.1% oxygen. Levels of gene expression relative to the calibrator sample were corrected for differences in the PCR efficiencies of the primers and probes used for each gene [ 10]. This entire procedure was repeated for blood samples drawn on three separate days from each of three CP patients and three age- and sex-matched normal controls.

### Physiology Protocols

For each participant, the experimental observations were made during the course of a single day. Participants reported to the laboratory at 8:30 A.M., where their air-breathing end-tidal partial pressure of oxygen ( Pet_O _2__) and carbon dioxide ( Pet_CO _2__) were measured, and venous and arterial blood samples were drawn. Each participant then undertook an identical series of four separate protocols, with 20 min separating each protocol. The first two protocols determined the participants' responses to very mild hypoxia, which approximated the level of hypoxia commonly experienced during commercial air travel. An initial 5 min of euoxia ( Pet_O _2__ of 100 mm Hg) was followed by 10 min of hypoxia at Pet_O _2__ of 70 mm Hg, which was then followed by a final 5 min of euoxia. The second two protocols followed an identical time course but determined responses to moderate hypoxia, using a stimulus Pet_O _2__ of 50 mm Hg rather than 70 mm Hg. This was close to the level of hypoxia experienced during acute exposure to an altitude of 3,500 m. Isocapnia was maintained throughout all four protocols, with Pet_CO _2__ maintained close to each participant's baseline value.

### Experimental Technique

Gas control during the four protocols was achieved using a technique known as dynamic end-tidal forcing, whereby a computer-controlled fast gas-mixing system allows the alveolar partial pressure of oxygen ( Po _2_) to be manipulated rapidly and accurately while at the same time maintaining alveolar partial pressure of carbon dioxide ( Pco _2_) constant [ 11]. Each protocol was conducted with the participant reclining in the left lateral position and breathing through a mouthpiece whilst wearing an occlusive nose clip. Ventilation was measured using a turbine volume-measuring device [ 12], and these data were averaged from the two protocols undertaken at each level of hypoxia. Respired gases were analysed continuously by mass spectrometry. These data were logged in real time to a personal computer that was at the same time controlling the end-tidal gas composition. A Hewlett-Packard (Palo Alto, California, United States) Sonos 5500 ultrasound machine with an S4 two-dimensional transducer (2–4 MHz) was used to perform continuous echocardiography. During the first protocol at each level of hypoxia, pulmonary vascular tone was assessed using a standard Doppler technique that has been extensively validated [ 13– 20]. In this technique, the maximum velocity of a regurgitant jet of blood through the tricuspid valve is measured during systole. The maximum systolic pressure gradient across the tricuspid valve (ΔP _max_) is then calculated beat by beat from this velocity using Bernoulli's equation. Right atrial pressure is unaffected by hypoxia [ 21], so changes in ΔP _max_ reflect changes in systolic pulmonary artery pressure. During the second protocol at each level of hypoxia, cardiac output was determined beat by beat using Doppler echocardiography to measure the velocity of systolic blood flow through the aortic valve [ 14, 15]. Heart rate was monitored by electrocardiography, and blood pressure was measured each minute using an automated cuff. Breath-by-breath measurements of pulmonary ventilation and beat-by-beat measurements of ΔP _max_, cardiac output, and heart rate were averaged over 1-min periods.

### Statistical Analyses

Specific differences between CP patients and normal controls were assessed statistically using Student's unpaired *t*-test (Microsoft Excel; Microsoft, Seattle, Washington, United States) and were considered significant at the *p* < 0.05 level. Other general comparisons of the effects of hypoxia were performed using a univariate repeated measures ANOVA, using the Greenhouse–Geisser correction to derive the degrees of freedom (SPSS statistical package; SPSS, Chicago, Illinois, United States).

## Results

The main results are presented as a comparison between the CP patient group and the age- and sex-matched normal healthy controls. The final section presents results from the polycythaemia control participants, as a comparison both with the normal controls and with the CP patients.

### Baseline Measurements

Results obtained from venous and arterial blood analyses are shown in Table 2. The arterial Pco_2_ was on average 6 mm Hg lower in the CP patients than in the matched normal controls ( *p* < 0.05). All CP patients had been chronically treated with venesection, and had normal haemoglobin and haematocrit with concomitant iron deficiency.

**Table 2:**
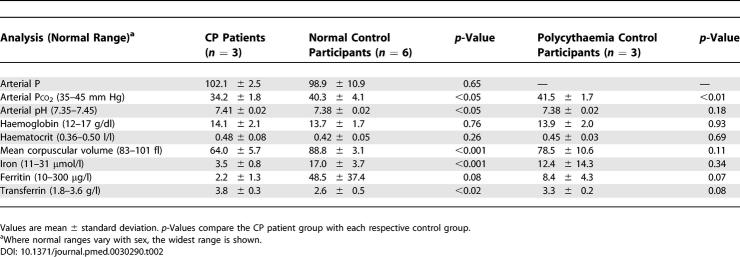
Arterial and Venous Blood Analyses

### Studies of Gene Expression

**Figure 1:**
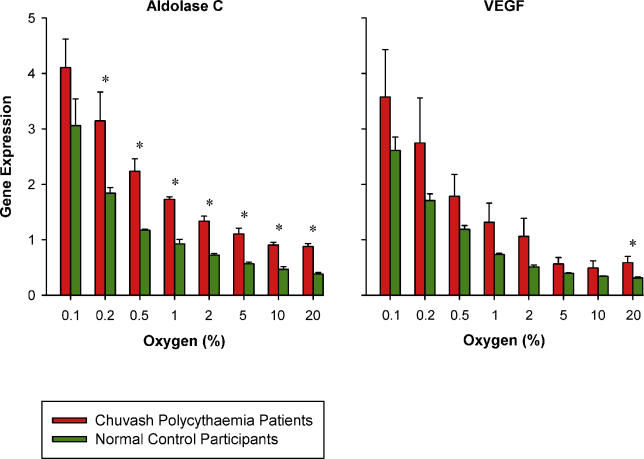
Aldolase C and *VEGF* mRNA Expression at Different Oxygen Tensions Lymphocytes were isolated from venous blood taken from CP patients and normal control participants, and incubated at eight different levels of oxygen tension prior to RNA isolation. Gene expression is shown relative to a standard calibrator sample. Basal gene expression at 20% oxygen was significantly higher in CP patients for both *Aldolase C* (A) and *VEGF* (B) ( *p* < 0.05). Both genes were induced by hypoxia, and at the lowest oxygen tension (0.1%) expression was no longer significantly different for either gene. Values are mean ± standard error of the mean. Asterisks indicate *p* < 0.05 (unpaired *t*-test).

Basal (non-hypoxic) expression of the HIF-regulated genes studied was significantly higher in the CP patient group ( *p* < 0.05; Figure 1). *Aldolase C* expression was 130% greater than in the controls, while *VEGF* expression was 90% greater. Both genes were induced by hypoxia, but at the most marked level of hypoxia, expression was no longer significantly greater in the CP patients. At this level of hypoxia, *Aldolase C* expression was only 34% greater than in the controls, and *VEGF* expression was only 37% greater. Thus, the effect of the VHL mutation was most striking under euoxic conditions, when the rate of hydroxylation of HIF-α is at its fastest.

### Pulmonary Ventilatory Measurements

**Figure 2:**
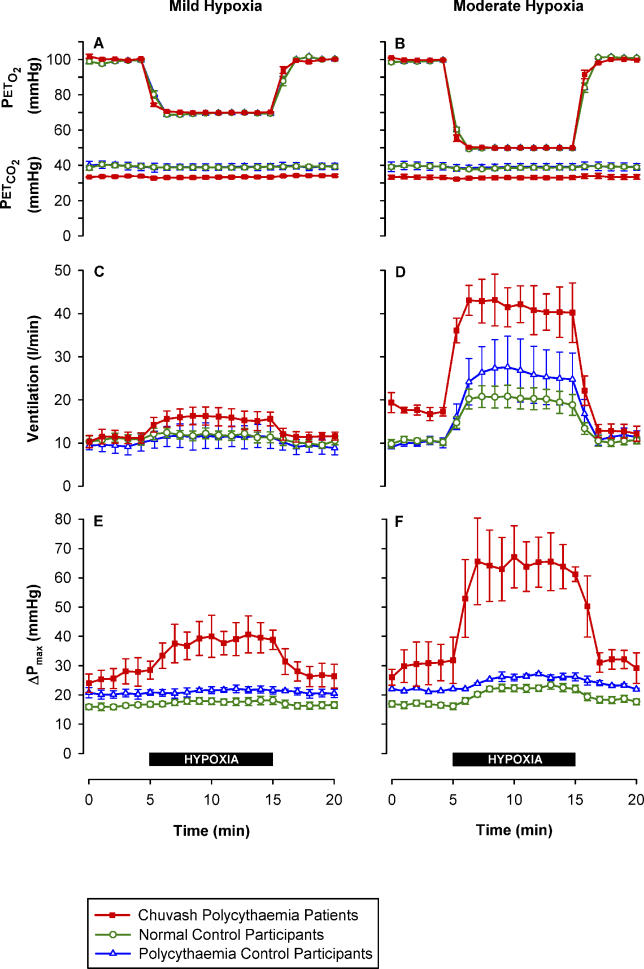
End-Tidal Gas Control, Ventilatory, and Pulmonary Vascular Responses to Mild and Moderate Hypoxia (A and B) End-tidal gas control. Pet _O _2__ and Pet_CO _2__ were well controlled. Pet _O _2__ was well matched between all groups. Pet_CO _2__ was lower in the CP patient group, reflecting this group's lower baseline air-breathing Pet_CO _2__. (C and D) Ventilation, given at body temperature and pressure, saturated with water vapour. Mild hypoxia provoked an increase in ventilation of 4.4 l/min in the CP patients versus 1.6 l/min in normal controls ( *p* < 0.05), while moderate hypoxia induced increases of 24.5 versus 10.0 l/min in these two groups, respectively ( *p* < 0.05). (E and F) Pulmonary vascular tone. This was assessed using Doppler echocardiography to determine ΔP _max_, a standard non-invasive index of pulmonary vascular tone. With mild hypoxia, ΔP _max_ increased by 11.5 mm Hg in the CP patient group compared with only 1.1 mm Hg in the normal control group ( *p* < 0.05). Moderate hypoxia stimulated a rise in ΔP _max_ of 35.3 mm Hg compared with 6.1 mm Hg in these two groups, respectively ( *p* < 0.001). All responses are shown during mild (A, C, and E) and moderate (B, D, and F) hypoxia. Values are mean ± standard error of the mean.

Figure 2 illustrates pulmonary ventilation at baseline and in response to hypoxia. During euoxia, ventilation was sometimes greater in the CP patient group than in the normal control group. Exposure to mild hypoxia provoked a 2.8-fold greater increase in ventilation in CP patients than in normal controls ( *p* < 0.02), in whom there was very little change in ventilation. For normal control participants, although ventilation increased more with moderate than mild hypoxia, all protocols were well tolerated. In contrast, the CP patients tolerated moderate hypoxia poorly, and one patient withdrew from the moderate hypoxia protocol with nausea and severe dyspnea. The rise in ventilation produced by moderate hypoxia was 2.4-fold greater in the CP patient group ( *p* < 0.05).

### Pulmonary Vascular Measurements

The CP patients displayed an abnormally high set point around which pulmonary arterial pressure was regulated, which was almost double that of the control group ( *p* < 0.005; Figure 2) and which, assuming a standard right atrial pressure of 5 mm Hg, exceeded the cut-off for pulmonary hypertension (pulmonary arterial pressure greater than 25 mm Hg) [ 22]. In normal control participants, as expected, exposure to mild hypoxia produced only a very small rise in ΔP _max_, reflecting their minimal pulmonary vasoconstrictive response. However, the increase in ΔP _max_ provoked by the same stimulus was 10.7-fold greater in the CP patient group ( *p* < 0.01). Their ΔP _max_ response to moderate hypoxia was likewise extremely abnormal, and was 5.8-fold greater than that for the normal control group ( *p* < 0.001).

### Systemic Vascular Measurements

**Figure 3:**
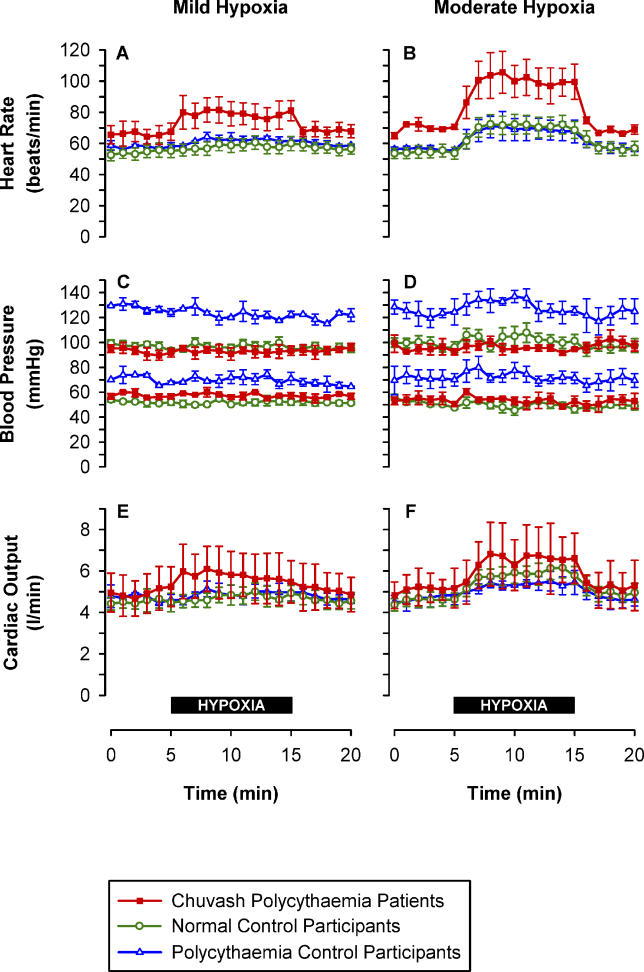
Systemic Vascular Responses to Mild and Moderate Hypoxia (A and B) Heart rate. Mild hypoxia provoked a rise of 11.7 beats/min in the CP patient group, compared with 3.6 beats/min in normal control participants ( *p* < 0.05). No other statistically significant differences in systemic vascular responses were detected between these two groups. (C and D) Blood pressure, showing systolic pressure (upper plot) and diastolic pressure (lower plot). (E and F) Cardiac output, assessed non-invasively using Doppler echocardiography. All responses are shown during mild (A, C, and E) and moderate (B, D, and F) hypoxia. Values are mean ± standard error of the mean.

There was a trend towards a higher baseline heart rate in CP patients than normal control participants ( *p* = 0.06; Figure 3), and upon transition to mild hypoxia, heart rate increased 3.2-fold more in the CP patient group ( *p* < 0.005); the corresponding difference was not statistically significant with moderate hypoxia (1.6-fold, *p* = 0.21). Changes in cardiac output followed a similar pattern to changes in heart rate but did not differ significantly between the two groups.

### Polycythaemia Control Participants

The polycythaemia control participants, like the CP patients, were iron-deficient with microcytic indices but normal haemoglobin and haematocrit ( Table 2). However, the polycythaemia control group, unlike the CP patient group, had a normal arterial Pco_2_ (significantly higher than that of the CP patient group, *p* < 0.01). Baseline pulmonary and systemic blood pressures were higher in the polycythaemia controls compared with the normal control group, but it should be noted that the polycythaemia control participants were older. The data from the polycythaemia control participant taking atenolol were excluded from the analyses of systemic vascular data.

**Figure 4:**
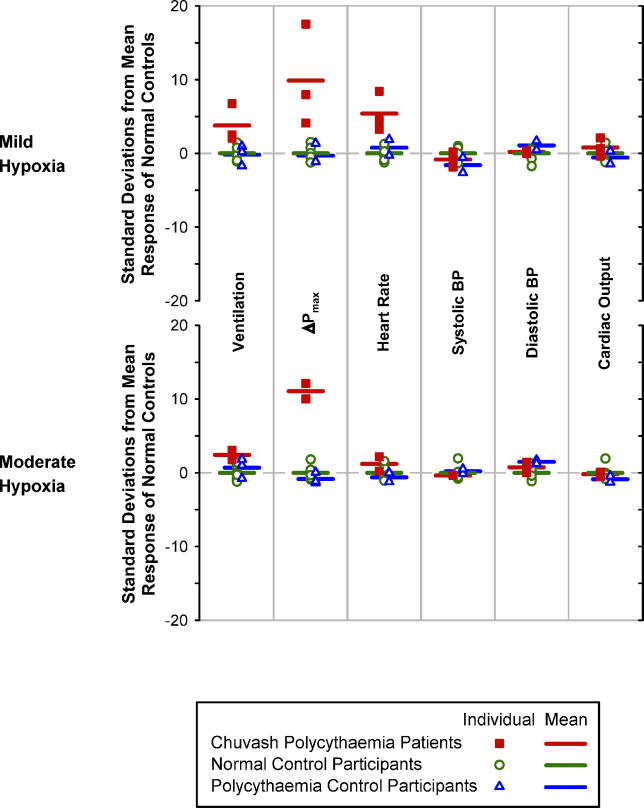
Sensitivities to Hypoxia for Individual Participants Results are shown in terms of the number of (normal control group) standard deviations by which each participant's response differed from the mean response of the normal control participants. The patients with CP were significantly different from the normal control group in their ventilatory and pulmonary vascular responses to both mild and moderate hypoxia, and in their heart rate responses to mild hypoxia. BP, blood pressure.

The mean responses (ventilation, ΔP _max_, heart rate, blood pressure, and cardiac output) of the polycythaemia control group to hypoxia are shown in Figures 2 and 3. In addition, Figure 4 illustrates the sensitivities to hypoxia of the individual participants from all three experimental groups. These are expressed in terms of the number of (normal control group) standard deviations that each participant's response deviated from the mean response of the normal healthy control participants. The responses of the polycythaemia control group never differed significantly from those of the normal control group (repeated measures ANOVA). This demonstrates that the major alterations in physiology present in the CP patients were not present in the polycythaemia control participants. As a final check on the overall results, the CP patient group was compared with the pooled control groups using repeated measures ANOVA. In all cases this analysis confirmed the significant findings reported in the preceding sections.

## Discussion

In CP the VHL–HIF signalling pathway is specifically and systemically impaired, uniquely facilitating human research into this transcriptional system. Previous phenotyping studies using transformed lymphocyte cell lines demonstrated increased basal expression of HIF-α with up-regulation of downstream target genes such as *EPO* and *VEGF* [ 8]. Using fresh lymphocytes, we have now confirmed that expression of classic HIF-regulated genes (of disparate functions) is increased under non-hypoxic conditions in CP patients. We have also shown that differences in gene expression are less distinct in hypoxia, consistent with a relative diminution in the impact of the mutant VHL when oxygen tension falls and the rate of hydroxylation of HIF-α slows. A retrospective matched cohort study conducted in Chuvashia found that homozygosity for the Chuvash VHL mutation was associated with vertebral haemangiomas, varicose veins, lower systemic blood pressures, and premature mortality partially related to cerebrovascular and thrombotic events [ 23]. The current study focuses on the phenotypic consequences of this mutation in systemic respiratory and cardiovascular physiology.

The lower air-breathing Pco_2_ measured in the CP patient group indicates an altered set point for respiratory control in the direction of an elevation in pulmonary ventilation (i.e., ventilation is somewhat higher than normal in relation to metabolism). This is consistent with measurements of their ventilation during isocapnic euoxia, which tended to be higher than for the controls although the difference was not statistically significant. It is striking that CP patients had both a reduced Pco _2_ set point for the respiratory controller and an abnormally high acute hypoxic ventilatory sensitivity, as together these are characteristic of individuals who are acclimatised to hypoxia at high altitude [ 24, 25]. Indeed, the difference in ventilatory sensitivity to hypoxia between CP patients and controls was likely to be even greater than reported, since the relative hypocapnia of the patients would have inhibited their ventilatory response [ 26]. These results are clearly consistent with the notion that the VHL–HIF system is involved in human ventilatory acclimatisation to hypoxia, and establish an important role for VHL in regulating respiratory control in hypoxia-naïve individuals.

CP patients were found to have a degree of pulmonary arterial hypertension. This most probably reflects HIF-regulated elevation in pulmonary vasomotor tone, although the increased rate of thrombotic events reported in CP [ 23] raises the theoretical possibility of chronic thromboembolic pulmonary hypertension as an alternative underlying mechanism. However, chronic thromboembolic pulmonary hypertension is rare even in patients with chronic uncontrolled myeloproliferative disease [ 27]. It is very unlikely to be present in young venesected individuals such as the patients in our study, who have no antecedent thrombotic history, hypoxaemia, or other clinical manifestations of thromboembolic disease. Furthermore, it should be noted that the presence of pulmonary hypertension in our patient group does not explain their very high pulmonary vascular sensitivity to hypoxia. A previous study measured pulmonary vasoresponsiveness to hypoxia in 26 eucapnic patients with pulmonary hypertension related to chronic obstructive lung disease [ 28]. For equivalent decrements in arterial Po _2_, the increase in pulmonary arterial pressures produced by hypoxia was 4–7 times smaller in the patients with chronic lung disease than in our CP patient group. Other studies using animals have also shown that hypoxic pulmonary vasoconstriction is blunted by increased pulmonary arterial pressures [ 29].

It is therefore remarkable that the CP patient group displayed such exquisite hypoxic pulmonary vascular sensitivity. Mild hypoxia provoked peak values for ΔP _max_ that correspond to systolic pulmonary arterial pressures of approximately 45 mm Hg, while the moderate hypoxic stimulus provoked extraordinary peak systolic pulmonary arterial pressures of approximately 70 mm Hg. If hypoxia were to be sustained, pulmonary arterial pressure might rise considerably higher, because in normal participants a second, gradual component of hypoxic pulmonary vasoconstriction develops [ 14] and continues to intensify for at least another 1–2 h [ 30]. Prolonged exposure to high pulmonary arterial pressures leads to right ventricular strain and eventual failure, introducing the possibility that pulmonary hypertension and exaggerated hypoxic sensitivity contribute to the premature mortality associated with CP [ 23]. This finding also raises the question of whether affected patients should avoid high altitudes and minimise long-haul air travel.

Lowered systemic blood pressure has previously been reported in CP [ 23], suggesting VHL–HIF may be involved in setting basal systemic vascular tone. Although no statistically significant differences in blood pressure were seen in our limited patient sample, our findings do implicate VHL–HIF in modulating the heart rate response to acute hypoxia.

In this study, it was clear that each individual with CP was physiologically very abnormal. There is no evidence to suggest that these pronounced physiological abnormalities were confounded by polycythaemia per se. Although relevant human data are lacking, animal studies have shown that the pulmonary vascular response to acute hypoxia is independent of haematocrit [ 31], and that the hypoxic ventilatory response is either inhibited [ 32] or unchanged [ 33] acutely by polycythaemia. In any case, none of the participants was polycythaemic at the time of the study, as therapeutic venesection had normalised the haemoglobin and the haematocrit in the affected patients.

Treatment of polycythaemia with venesection renders patients iron-deficient. Iron is an obligate co-factor in the prolyl hydroxylation reaction by which HIF is regulated [ 4, 5], and iron deficiency inhibits this reaction, at least in vitro. Thus, it is possible that, through effects on HIF-regulated gene expression, iron deficiency contributed to the physiological abnormalities seen in the CP patients. Our study did not detect any significant physiological differences between the polycythaemia control group, who were clearly iron-deficient, and the normal controls. This tends to suggest that associated iron deficiency is not a factor underlying the abnormalities observed within the CP patient group. It should nevertheless be noted that the polycythaemia control participants were not as severely iron-deficient as the CP patients, and also that there was some suggestion that the ventilatory response to moderate (but not mild) hypoxia of the polycythaemia control group was intermediate between that of the normal control and CP groups. Thus, while we were unable to show that the iron deficiency present in polycythaemia control participants resulted in abnormal cardiopulmonary physiology, the number of participants studied was small and the possibility clearly remains that iron deficiency may phenocopy VHL loss of function to some limited extent.

One of the polycythaemia control participants was taking atenolol. Results from this participant were excluded from the analysis of systemic vascular data but were included in all other analyses. Ventilatory responses to acute hypoxia are not affected by β-adrenergic receptor blockade [ 34, 35], and animal studies suggest that β-receptor antagonists either have no effect on hypoxic pulmonary vasoconstriction [ 36] or enhance the phenomenon somewhat [ 37].

Overall, the results from the polycythaemia control participants support the notion that the cardiopulmonary abnormalities in CP result directly from dysregulation of the VHL pathway rather than indirectly from the haematological manifestations of the disease.

The major known role of VHL is its regulation of the HIF transcriptional system [ 2]. The results from the present study and others [ 8] demonstrate that the Chuvash VHL mutation results in up-regulation of at least some HIF-responsive genes under conditions of euoxia. Furthermore, a role for the HIF system in cardiopulmonary regulation has been observed through the attenuation of cardiopulmonary responses to hypoxia in mice that are heterozygously deficient for functional HIF genes [ 38– 41]. This down-regulation of cardiopulmonary responses in HIF-α ^+/−^ mice is consistent with the up-regulation of cardiopulmonary responses seen in the CP patients in this study. It thus seems likely that the phenotype observed in the current study results from effects of VHL on the HIF system, though the involvement of other (as yet unknown) proteins with which VHL may interact cannot be excluded.

Involvement of VHL–HIF in vasomotor regulation is consistent with the nature of some of the genes HIF is known to regulate in humans and animals. These genes include *endothelin-1, endothelial nitric-oxide synthase, tyrosine hydroxylase, α _1β_-adrenergic receptor, adrenomedullin, heme oxygenase-1,* and *atrial natriuretic peptide* [ 42]. Tyrosine hydroxylase has also been hypothetically linked to ventilatory control through its regulation of catecholamine biosynthesis in the carotid body [ 43]. In addition, transgenic mice over-expressing EPO in the brain were recently shown to have abnormal ventilatory responses to hypoxia [ 44], raising the further possibility that the VHL–HIF pathway influences ventilation through regulating cerebral expression of EPO.

Although the number of patients in our study was small, and as such our findings may not be completely representative of CP patients overall, our findings nevertheless demonstrate that a relatively subtle disorder of HIF degradation profoundly alters human cardiopulmonary physiology. Patients with CP had elevated basal ventilation and pulmonary vascular tone, with extremely high ventilatory, pulmonary vasoconstrictive, and heart rate responses to acute hypoxia. The abnormalities they displayed mimicked those caused by acclimatisation to hypoxia at high altitude. We conclude that, in humans, the VHL–HIF transcriptional signalling pathway, which is central to intracellular oxygen sensing, also appears to play a major role in calibrating the organ systems upon which cellular oxygen delivery ultimately depends. Further research is required to investigate the exact mechanisms involved. Our study also has implications for current HIF-related therapeutic research, much of which is directed towards pharmacological inhibition of the prolyl hydroxylases that negatively regulate HIF. Such therapies are intended to up-regulate HIF-dependent genes and promote angiogenesis in ischaemic/hypoxic vascular disease, but will now require surveillance for the undesired physiological effects of exaggerated hypoxic responses and elevated pulmonary vascular tone. Conversely, HIF inhibitors under investigation as anti-cancer treatments may have opposite physiological effects, conceivably introducing potential applications for these agents in diseases such as pulmonary hypertension.

## Abbreviations

ΔP _max_: maximum systolic pressure gradient across the tricuspid valve (a standard echocardiographic index of pulmonary vascular tone)
CP: Chuvash polycythaemia
EPO: erythropoietin
HIF: hypoxia-inducible factor
Pet_CO _2__: end-tidal partial pressure of carbon dioxide
Pet_O _2__: end-tidal partial pressure of oxygen
Pco_2_: partial pressure of carbon dioxide
Po_2_: partial pressure of oxygen
VEGF: vascular endothelial growth factor
VHL: von Hippel–Lindau tumour suppressor protein
